# Telemedicine and Resource Utilization in Pulmonary Clinic

**DOI:** 10.1186/s12890-024-03066-x

**Published:** 2024-06-05

**Authors:** Rose M Puthumana, Claire A Grosgogeat, Jenna K Davis, Linda V Bocanegra, Samira Patel, Tanira Ferreira, Dipen J Parekh, Hayley B Gershengorn, Abigail L Koch

**Affiliations:** 1 Department of Internal Medicine, University of Miami and Jackson Health Systems, 1611 NW 12th Ave, 1569 NW 17th Ave, Apt 1005, Miami, FL 33136, 33125 United States of America; 2 University of Miami Miller School of Medicine, 1600 NW 10th Ave, Miami, FL 33136 United States of America; 3 Division of Pulmonary, Critical Care, and Sleep Medicine, University of Miami and Jackson Health Systems, 1600 NW 10th Ave, Miami, FL 33136 United States of America; 4 University of Miami Hospitals and Clinics, 1400 NW 12th Ave, Miami, FL 33136 United States of America; 5 Division of Pulmonary, Critical Care, and Sleep Medicine, University of Miami Miller School of Medicine, 1600 NW 10th Ave, Miami, FL 33136 United States of America; 6 Department of Urology, University of Miami Miller School of Medicine, 1600 NW 10th Ave, Miami, FL 33136 United States of America; 7 Albert Einstein College of Medicine, Division of Critical Care Medicine, 1300 Morris Park Ave, The Bronx, NY 10461 United States of America

**Keywords:** Telehealth, Virtual medicine, Pulmonary medicine, Outpatients, Delivery of health care, Health resources

## Abstract

**Background:**

Telemedicine use increased with the Covid-19 pandemic. The impact of telemedicine on resource use in pulmonary clinics is unknown.

**Methods:**

This retrospective cohort study identified adults with pulmonary clinic visits at the University of Miami Hospital and Clinics (January 2018-December 2021). The primary exposure was telemedicine versus in-person visits. Standard statistics were used to describe the cohort and compare patients stratified by visit type. Multivariable logistic regression models evaluated the association of telemedicine with resource use (primarily, computed tomography [CT] orders placed within 7 days of visit).

**Results:**

21,744 clinic visits were included: 5,480 (25.2%) telemedicine and 16,264 (74.8%) in-person. In both, the majority were < 65-years-old, female, and identified as Hispanic white. Patients seen with telemedicine had increased odds of having CT scans ordered within 7 days (adjusted odds ratio [aOR] 1.34, [95% confidence interval 1.04–1.74]); and decreased odds of chest x-rays (aOR 0.37 [0.23–0.57]). Telemedicine increased odds of contact of any kind with our healthcare system within 30-days (aOR 1.56 [1.29–1.88]) and 90-days (aOR 1.39 [1.17–1.64]). Specifically, telemedicine visits had decreased odds of emergency department visits and hospitalizations (30 days: aOR 0.54 [0.38–0.76]; 90 days: aOR 0.68 [0.52–0.89]), but increased odds of phone calls and electronic health record inbox messages (30 days: aOR 3.44 [2.73–4.35]; 90 days: aOR 3.58 [2.95–4.35]).

**Conclusions:**

Telemedicine was associated with an increased odds of chest CT order with a concomitant decreased odds of chest x-ray order. Increased contact with the healthcare system with telemedicine may represent a larger time burden for outpatient clinicians.

**Supplementary Information:**

The online version contains supplementary material available at 10.1186/s12890-024-03066-x.

## Background

The Covid-19 pandemic made the usage of telemedicine mainstream and acceptable in healthcare [1, 2]. During the height of the pandemic, telemedicine was vital to providing essential healthcare services to patients most at risk of serious complications from Covid-19 [3]. Telemedicine has persisted due to the perceived benefits including increased efficiency, improved access to care, and cost-savings for the patient (less travel and time away from work) and, potentially, healthcare organizations [4, 5]. While patient attitudes regarding telemedicine are generally positive, citing increased feelings of autonomy and security [6–8], existing research fails to correlate these sentiments with improvements in quality of life [9]. In addition, patients and providers alike report concerns about the lack of a complete physical exam in the telemedicine setting [10]. If and how providers may adapt to the diagnostic barriers presented by the lack of physical exams over telemedicine, for example by ordering more imaging studies, is unknown.

Evaluation of telemedicine outcomes has focused mainly on the use of telemonitoring– virtual surveillance visits in addition to standard in-person visits– rather than comparing telemedicine visits directly to in-person visits [11]. Previous literature proposes that telemonitoring may correlate with decreased emergency department (ED) visits and readmissions for pulmonary patients [12]. Notably, this was not ubiquitous across all studies in pulmonary patients, and mortality with telemedicine tended to be unchanged or increased for COPD patients [13, 14]. The literature is more limited on the impact of telemedicine visits as replacement for in-person visits with providers. Few studies assess diagnostic decisions, such as imaging orders, between telemedicine and non-telemedicine patients, and the studies that do exclude patients with chronic conditions or choose to focus on a singular diagnosis [15, 16]. Chest CT is a high value exam in the pulmonary clinic and often ordered following a chest x-ray if the chest x-ray is non-diagnostic or does not provide the detail needed for planning. CT scans are more costly, but provide significant information to the pulmonologist. Some CT findings such as pneumonia, edema, or interstitial lung disease may be elicited via pulmonary physical exam. In the absence of an in person encounter and ability to complete a physical exam, chest CT may serve as a costly surrogate.

In this retrospective cohort study, we sought to evaluate imaging ordering practices during telemedicine visits for a heterogenous cohort of pulmonary patients by comparing telemedicine appointments to in-person appointments. We hypothesized that telemedicine visits would be associated with an increased resource utilization, in particular, advanced imaging such as chest computed tomography (CT). Such an increase in resource use would be important to acknowledge in the context of telemedicine persistence in the post-pandemic era.

## Materials and Methods

We identified adults with pulmonary clinic visits at the University of Miami Hospital and Clinics, both through telemedicine and traditional in-person clinic visits, from January 2018 to December 2021. Telemedicine visits were accessed through the Electronic Health Record (EHR) and utilized integrated Zoom Video Communications (San Jose, CA) software. The use of a device supporting Zoom was required to participate in a telemedicine visit. The choice to utilize telemedicine was determined using shared decision making between providers and patients, it was never mandated by the institution. Patients whose visits were via phone call only or were completed by nonphysician providers, providers who did not see patients in each of the 4 study years, or providers who saw fewer than 5 pulmonary patients per year were excluded (supplemental Fig. 1). The exclusion of these scenarios was made to ensure similar practice patterns as much as possible, based on type of training. All included providers were physicians with sub-specialty training in pulmonary medicine.

The primary exposure was telemedicine versus in-person visits; where physicians and patients determined how often an in-person visit would be held between tele-health visits in a pragmatic manner. The primary outcome, selected a priori, based on its significant diagnostic value as well as cost in the pulmonary clinic setting, was chest CT orders placed within 7 days following the clinic visit. Because in this institution providers are allowed 7 days to place an order within an outpatient encounter; we analyzed a 7-day period to ensure that all orders associated with the encounter were captured. Secondary outcomes included chest x-ray, echocardiogram, and pulmonary function tests (PFTs) ordered within 7 days, follow up appointments via telemedicine or in-person within 30 and 90 days, ED visits within 30 and 90 days, EHR inbox messages, or telephone encounters within 30 and 90 days.

Baseline characteristic data were collected from the EHR including demographics, comorbidities, and pulmonary diagnoses. Patient encounters were assigned to one or more of ten diagnosis subgroups (based on International Classification of Diseases, 10th Revision codes in the EHR, supplemental Table 1): abnormal imaging/PFT/arterial blood gas (ABG), cystic fibrosis (CF) and congenital lung diseases, deconditioning and dyspnea, pleural disease, respiratory failure and mechanical ventilation, interstitial lung disease (ILD), lung nodules and masses, obstructive lung disease, pulmonary hypertension (PH) and cardiopulmonary disease, and tobacco use disorder. For patients with PFT data in the EHR, PFT data were collected from the test closest in time to the clinic visit; from these, the presence of obstruction was determined (FEV1/FVC < 70%) [17], as well as the median forced expiratory volume in one second (FEV1), forced vital capacity (FVC), diffusing capacity of the lungs for carbon monoxide (DLCO) and residual volume (RV) for both cohorts. For each encounter, prescribed medications were identified and categorized as: non-steroid inhalers, steroid inhalers, oral steroids, ILD medications, CF medications, PH medications, biologics, and other. The number of clinic visits (telemedicine or in-person) and, separately, the number of hospital admissions at our institution within the past year were collected as well.

We used standard summary statistics to describe the cohort and Chi-square or Wilcoxon rank sum testing as appropriate to compare baseline characteristics and unadjusted outcomes between patient encounters via telemedicine vs. in-person. To evaluate the independent association of being seen in a telemedicine vs. an in-person visit with our primary outcome, 7-day CT scan orders, we constructed a multivariable logistic regression model including the following *a priori* selected covariables: age (< 65, 65–74, 75+); gender; race/ethnicity (non-Hispanic White, non-Hispanic Black, Hispanic White, Hispanic Black, other, unknown); primary insurance provider (commercial, Medicaid, Medicare); distance of home zip code from hospital zip code (≤ 5, > 5–10, > 10–15, > 15–20, > 20 miles or unknown); number of Elixhauser comorbidities [18] (modeled continuously); body mass index (< 25, 25-34.9. 35+, unknown); smoking status (never, former, current/time unknown, unknown); number of pulmonary clinic (telemedicine or in-person) visits in the prior year (modeled continuously); and number of hospitalizations in the prior year (modeled continuously). We then created similar models for each secondary outcome. These covariables were selected a priori based on clinical judgment specifically because they may influence access to care and severity of disease.

To evaluate the association of telemedicine use with 7-day CT scan orders for individual disease states, we recreated the primary model for each pulmonary diagnosis subgroup separately. We also created an additional model for two of the subgroups including relevant PFT data: [1] ILD patients including pre-bronchodilator FVC and [2] obstructive lung disease patients including pre-bronchodilator FEV1. Post-bronchodilator data was not available for the majority of patients. Obstruction was defined as FEV1/FVC < 70% [17]. As PFT data were missing in a large number of patients, we created these additional models in two ways: including only complete cases and with multiple imputation for missing PFT values (with 10 imputations). Finally, as a sensitivity analysis aimed at minimizing bias related to which patients were seen using telemedicine vs. in-person once telemedicine was available, we reconstructed the primary model for a restricted cohort of patients including those seen in in-person visits in 2018–2019 and those seen via telemedicine in 2020–2021.

All analyses were performed using STATA/MP 17 (StataCorp, College Station, Texas) and Microsoft Excel (Microsoft, Redmond, Washington) in accordance with an a priori derived statistical analysis plan. P-values < 0.05 were considered significant; no adjustment was made for multiple comparisons; thus all non-primary analyses should be considered hypothesis generating. The study was IRB approved, ID: 20,200,739.

## Results

A total of 21,744 clinic visits were included. 5,480 (25.2%) visits were via telemedicine and 16,264 (74.8%) of the visits were in-person visits (Table 1). Of the 21,744 visits, 4,195 were initial visits for the patient. There were 8,738 unique patients seen during the study period (supplemental Table 2, supplemental Fig. 2). The majority of the cohort was < 65 years of age. Over half the sample identified as Hispanic white. Most of the sample’s payor source was commercial insurance. The telemedicine cohort lived further from the clinic and had fewer comorbidities. The most frequently identified comorbidities were chronic lung disease, hypertension, obesity, solid tumors, and iron deficiency anemia (supplemental Table 3). In the telemedicine sample, 55.7% identified as never smoking, 37.4% identified as formerly smoking, 4.9% identified as currently smoking, and 2.0% had missing data. Within the in-person visit sample, 56.5% identified as never smoking, 38.9% identified as formerly smoking, 4.4% were currently smoking, and 0.2% had missing data.

**Table 1:** Cohort characteristics

	*In-Person Visits, N(%)*	*Telemedicine, N(%)*	*p-value*
*Number of visits, N(%)*	16,264 (74.8)	5,480 (25.2)	
*General Characteristics*			
*Age*			0.16
<65	9,362 (57.6)	3,224 (58.8)	
65–74	4,118 (25.3)	1,373 (25.1)	
75+	2,784 (17.1)	883 (61.7)	
*Gender*			0.16
Female	9,859 (60.6)	3,381 (61.7)	
Male	6,405 (39.4)	2,099 (38.3)	
*Race/Ethnicity*			< 0.001
Non-Hispanic White	4,181 (25.7)	1,570 (28.6)	
Non-Hispanic Black	2,990 (12.2)	497 (9.1)	
Hispanic White	8,580 (52.6)	2,902 (53.0)	
Hispanic Black	294 (1.8)	86 (1.6)	
Other	639 (3.9)	213 (3.9)	
Unknown	580 (3.6)	212 (3.9)	
*Body Mass Index (BMI)*			< 0.001
<25	5,358 (32.9)	41 (0.7)	
25-34.9	5,109 (31.4)	46 (0.8)	
35+	5,290 (32.5)	29 (0.5)	
*Payor Source*			0.013
Commercial	11,631 (71.5)	4,025 (73.4)	
Medicaid	111 (0.7)	28 (0.5)	
Medicare	4,522 (27.8)	1,427 (26.0)	
*Comorbidities*			
Number of Elixhauser Comorbidities, Median (inter-quartile range)	3	2	< 0.001
*Smoking*			< 0.001
Never	9,194 (56.5)	3,052 (55.7)	
Former	6,323 (38.9)	2,048 (37.4)	
Current/Duration Unknown	715 (4.4)	270 (4.9)	
Unknown	32 (0.2)	110 (2.0)	
*Number of Clinic Visits in Past 1 Year*			< 0.001
0	7,102 (43.7)	2,194 (40.0)	
1	4,130 (25.4)	1,513 (27.6)	
2	2,216 (13.6)	783 (14.3)	
3+	2,816 (17.3)	990 (18.1)	
*Number of Hospital Admissions in Past 1 Year*			0.016
0	14,027 (86.2)	4,803 (87.6)	
1	1543 (9.5)	493 (9.0)	
2	410 (2.5)	109 (2.0)	
3+	284 (1.7)	75 (1.4)	

Table displaying cohort demographics and characteristics

The number of patients with more than one diagnosis was determined for each category (supplemental Table 4). Of the 10 diagnosis subgroups, obstructive lung disease was the most represented (39.1% telemedicine, 38.1% in-person) (Table 2). The telemedicine sample had a higher percentage of encounters for abnormal imaging/PFT/ABG (33.7% vs. 25.7%), CF and congenital disease (7.2% vs. 5.7%), and lung nodules (30.8% vs. 20.2%). The in-person sample had more encounters for ILD (19.8% vs. 17.8%) and PH/cardiopulmonary disease (8.9% vs. 4.8%). Of the patients with obstruction detected on PFT, 12.7% were seen via telemedicine (supplemental Fig. 3). Of those with severe obstruction, 24.4% utilized telemedicine compared to 4.4% of those with mild obstruction.

**Table 2:** Diagnosis Subgroups and Medications Used

	*In-Person Visits, N(%)*	*Telemedicine, N(%)*	*p-value*
Diagnosis Subgroup*			
Abnormal Imaging/ Pulmonary Function Testing (PFT)/ABG (Arterial Blood Gas)	3,329 (25.7)	1,530 (33.7)	< 0.001
Cystic Fibrosis (CF)/ Congenital Lung Disease	732 (5.7)	325 (7.2)	< 0.001
Deconditioning/Dyspnea	1,947 (15.1)	667 (14.7)	0.58
Pleural Disease	250 (1.9)	91 (2.0)	0.76
Hypoxemia/Acute Respiratory Failure (RF)/Ventilator/Tracheostomy	387 (3.0)	142 (3.1)	0.64
Interstitial Lung Disease (ILD)	2,567 (19.8)	806 (17.8)	0.002
Nodules/Masses	2,617 (20.2)	1,395 (30.8)	< 0.001
Obstructive Disease	4,934 (38.1)	1,771 (39.1)	0.28
Pulmonary Hypertension (pHTN)/ Cardiopulmonary Disease	1,146 (8.9)	216 (4.8)	< 0.001
Tobacco Use	327 (2.5)	98 (2.2)	0.17
*Medications*			
Non-Steroid Inhalers	4,272 (79.8)	1,074 (75.5)	0.001
Steroid Inhalers	491 (9.2)	334 (23.5)	< 0.001
Oral Steroids	1,082 (20.2)	250 (17.6)	0.027
ILD Medications	47 (0.9)	2 (0.1)	0.004
CF Medications	26 (0.5)	46 (3.2)	< 0.001
pHTN Medications	1,146 (8.9)	216 (4.8)	< 0.001
Biologics	14 (0.3)	1 (0.1)	0.17
Other Medications	4 (0.1)	4 (0.3)	0.044

Diagnosis Subgroups and Medications Used

* Values sum to > 100% as patients may receive more than one diagnosis

Among both visit types, the majority of patients were prescribed non-steroid inhalers (75.5% of telemedicine, 79.8% in-person). A greater percentage of the telemedicine cohort was prescribed steroid inhalers (23.5% vs. 9.2%). Oral steroids were prescribed to a higher percentage of the in-person cohort (20.2% vs. 17.6%). The majority of patients in both groups had at least one clinic visit within the past one year, but no hospitalizations. In both the telemedicine and in-person samples, a minority of patients had completed PFTs (6.7% vs. 13.8%, Table 3). Of those with completed PFTs, 26.4% of the in-person and 23.3% of the telemedicine sample had evidence of obstruction.

**Table 3:** Pulmonary Function Test Summary

	*In-Person Visit, N(%) or median (IQR)*	*Telemedicine Visit, N (%) or* *median (IQR)*	*p-value*
*#PFTs, N (% of all patients)*	2,244 (13.8)	367 (8.7)	
*PFT Findings* ^†^			
Obstruction (FEV1/FVC < 70%), N (%)	585 (26.4)	85 (23.3)	0.21
FEV 1, median (IQR)	77 (62,91)	77 (61,92)	0.97
FVC, median (IQR)	81 (67,94)	80 (66,93)	0.19
DLCO, median (IQR)	65 (48,82)	65 (50,80)	0.83
RV, median (IQR)	59 (41,80)	63 (45,83)	0.46

Table displaying # of patients with completed PFTs, presence of obstruction, and median values for selected PFT measurements

†partial PFT data on 2,611 (12.0% of cohort). Missing data from 2,611 patients on: obstruction: 394 (1.1%), FEV1: 14 (0.5%), FVC: 10 (0.4%), DLCO: 1756 (67.3%), RV: 2051 (78.6%). Obstruction defined as pre-FEV1/FVC < 70% (post only available for 210 patients)

**Fig. 1:**
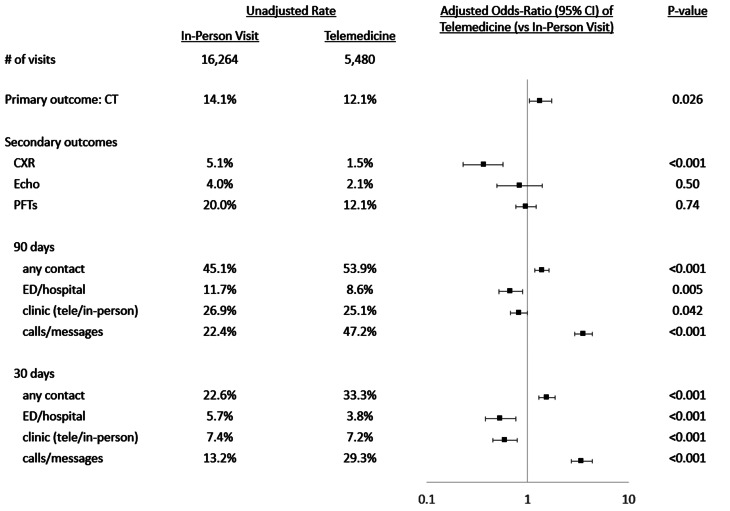
Association of Telemedicine with Resource Use. Figure displaying unadjusted and adjusted odds ratios of each outcome with the use of telemedicine vs. in-person visit. CT: chest computed tomography; CXR: chest x-ray; Echo: echocardiogram; PFT: pulmonary function test; ED: emergency department; tele: telemedicine

In unadjusted analyses, within the telemedicine group, 12.1% of patients had a CT scan ordered within 7 days compared to 14.1% of patients in the in-person group (Fig. 1). The telemedicine group had a lower unadjusted rate of chest x-ray (1.5% vs. 5.2%), echocardiogram (2.1% vs. 4.0%), and PFT (12.1% vs. 20%) orders within 7 days of clinic visit. Patients seen via telemedicine had increased unadjusted rates of any contact with the healthcare system both within 30 days (53.9% vs. 45.1%) and 90 days (33.3% vs. 22.6%).

After adjustment for planned covariables as noted in methods, there was an increased odds of CT scans ordered within 7 days of their clinic visit for patients seen via telemedicine (adjusted odds-ratio [aOR] 1.34 [95% confidence interval 1.04–1.74]. In contrast, patients seen via telemedicine had a decreased odds of chest x-ray orders (aOR 0.37, [0.23–0.57]). There was no association was found between echocardiogram or PFT orders and the usage of telemedicine. Telemedicine clinic visits were associated with increased contact of any kind with the healthcare system both within 30 days (aOR 1.56 [1.29–1.88]) and 90 days (aOR 1.39 [1.17–1.64]) which was driven by an increased odds of phone calls and EHR inbox messages (in 30 days aOR 3.44 [2.73–4.35]; in 90 days aOR 3.58 [2.95–4.35]). Notably, telemedicine was associated with a decreased odds of ED visits and hospitalizations at both 30 days (aOR 0.54 [0.38–0.76]) as well as 90 days (aOR 0.68 [CI 0.52–0.89]. There was no association between telemedicine usage and follow up clinic visits (both telemedicine and in-person) both within 30 and 90 days. To account for the Covid-19 pandemic specific practice patterns, Sensitivity analysis limiting the telemedicine cohort to encounters in 2020–2021 and the in-person cohort to encounters in 2018–2019 yielded similar results to the primary analysis (supplemental Fig. 4).

**Fig. 2:**
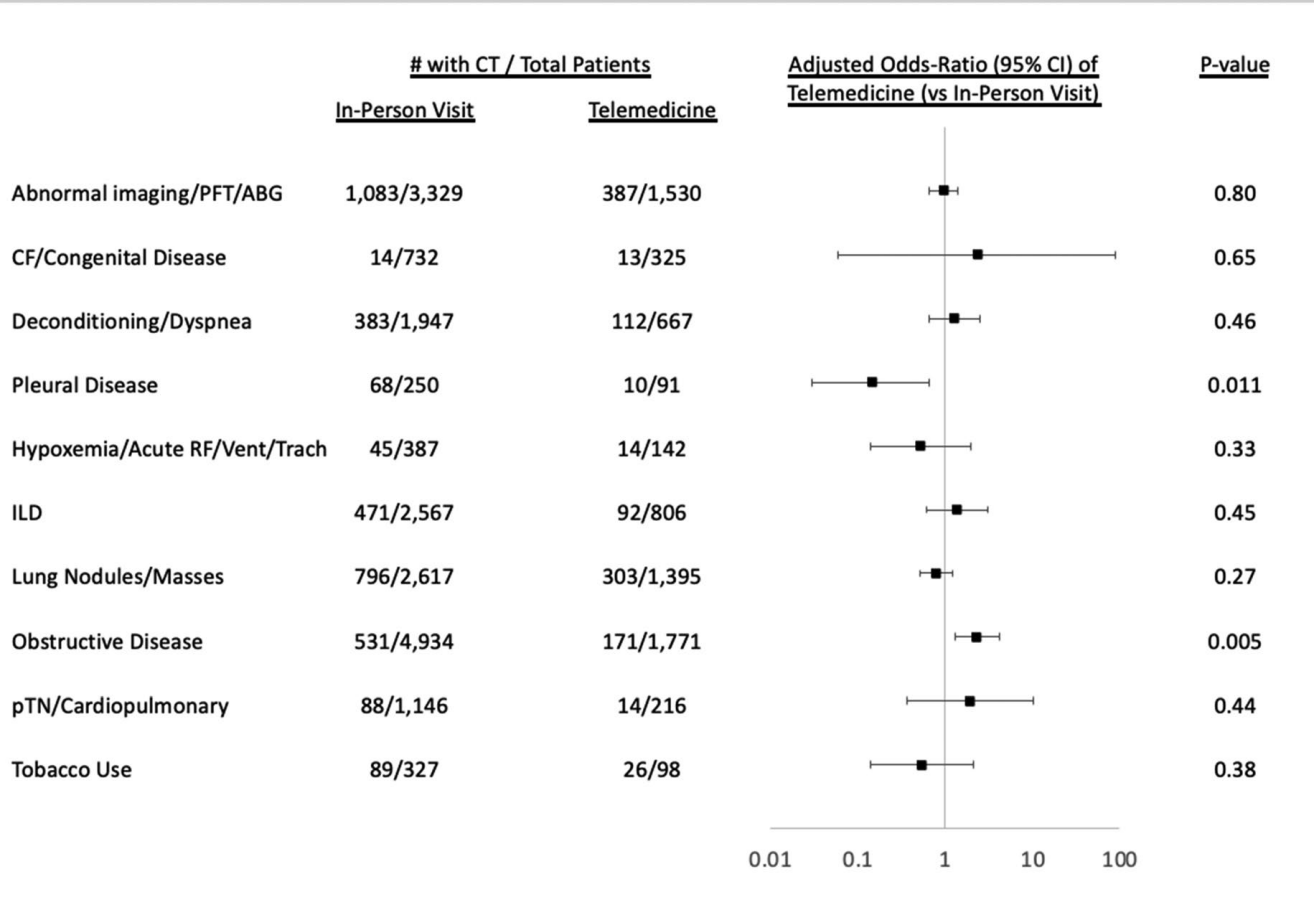
Association of Telemedicine with CT Use Stratified by Diagnosis. Figure displaying unadjusted and adjusted odds ratios of CT scan order for each diagnosis subgroup with the use of telemedicine vs. in-person visit. CT: chest computed tomography; ABG: arterial blood gas; PFT: pulmonary function test; CF: cystic fibrosis; RF: respiratory failure; Vent: ventilator associated; Trach: tracheostomy associated; ILD: interstitial lung disease; pHTN: pulmonary hypertension

When patients were stratified by diagnosis, patients with obstructive lung disease who were seen via telemedicine had an increased odds of CT scan order (aOR 2.32 [1.29–4.20]). In contrast, the group with diagnoses including hemothorax, pneumothorax, and pleural effusions, who were seen via telemedicine had a decreased odds of CT scan order (aOR 0.15 [0.03–0.65]) (Fig. 2). Inclusion of PFT data in the models did not alter the findings: patients with obstructive disease had an increased odds of CT scan order when seen via telemedicine (complete cases: aOR 1.84 [0.45–7.49]; imputation: aOR 2.36 [1.3–4.27]; supplemental Table 5); patients with ILD did not.

## Discussion

Telemedicine and virtual visits were rapidly integrated into existing clinic models during the Covid-19 pandemic, with unclear impacts on pulmonary physician ordering practices and resource use. In our study, being seen via telemedicine for pulmonary medicine clinic was associated with increased odds of chest CT scans being ordered and decreased odds of chest x-rays when adjusted for factors that may indicate disease severity and access to care. Together, these findings suggest that when patients are seen via virtual visits, where providers have less objective data available (i.e., unable to complete a physical exam), higher value imaging that offers more data is ordered.

Furthermore, telemedicine visits were associated with increased overall contact with the healthcare system driven, particularly, by the increased usage of follow up calls and messages. It is possible that some degree of this increased communication may result from unmeasured confounding as patients who opted to use telemedicine may be more comfortable with technology and digital communication, and therefore also utilized Epic messaging more frequently. Also possible, however, is that not seeing their providers in-person left patients with more of a need for further contact. In this era, a hybrid method of in-person visits where a physical exam could be completed intermixed with telehealth visits may prove to be the best use of resources; further study is required to identify the most effective way to hybridize these modalities.

While total contact with the healthcare system was increased, ED visits, hospitalizations, and close clinic follow up through either telemedicine or in-person formats, was decreased with telemedicine usage. When resource utilization in telemedicine compared to in-person clinic visits has previously been studied, the focus is often on total monetary cost [19–23], and few of these studies have looked at pulmonary clinics specifically. While the direct costs to the institution of maintaining and staffing an in-person clinic are significant, our study showed that there may be less obvious financial costs to the patient and institution that may arise with the use of telemedicine in pulmonary clinics. Increased contact with the healthcare system, primarily in the form of phone calls and messages, can be costly in terms of time for the provider. Understanding how this cost balances against the cost required for potentially reduced hospitalizations and ED/clinic visits is complex (e.g., different providers likely tend to use phone calls/messages rather than see patients in the clinic) and requires further study. Moreover, the increased use of CT scans and decreased use of chest X-rays also results patient-centered cost financially as well as an increased dose of radiation exposure [24]. 

Existing literature regarding telemedicine specifically in pulmonary clinics has largely focused on whether usage may be beneficial to patients and/or hospital systems by preventing ED and inpatient admissions [7, 14, 25–31]. However, these studies were heterogenous in their findings and often focused on a sub-group of pulmonary patients such as only those with COPD [3, 9, 28, 29, 32–39] or lung cancer [40–43]. In addition, existing studies were completed prior to the Covid-19 pandemic and mostly used telemedicine to augment in-person visits through monitoring systems rather than as a form of outpatient encounter [26–31, 37–39, 44–50]. Those evaluating the use of telemedicine as a replacement for in-person clinic visits, particularly during the Covid-19 pandemic, focused on patient sentiments rather than objective measures [32, 33, 41, 42]. Our analysis of resource utilization with focus on diagnostic testing is unique.

Stratifying the study population by diagnosis and by lung function showed that specific groups may be differentially suited to telemedicine. In patients with obstructive lung disease, a higher percentage of the those with severe obstruction were seen via telemedicine compared to those with lesser degrees of obstruction. This finding reinforces existing research suggesting that this pattern may be due to either provider or patient preference to mitigate infection risk or reduce the burden of travel [3, 4] for patients with severe obstruction. However, we found that patients with obstructive lung disease had an increased odds of CT scan order when seen via telemedicine vs. in-person. When choosing telemedicine for this population, therefore, perceived benefits of decreased risk of infection or reduced travel burden will need to be balanced with the increase in odds of CT scan orders and known lack of improvement in quality of life [9] or proven efficacy in reducing ED visits and readmissions [27]. 

Our study has limitations. In terms of design, our study is retrospective, so the collection of all demographic and clinical factors is limited by the accuracy of medical documentation. Further, as with any retrospective study, there remains the possibility of residual confounding. In addition, chronic lung disease was one of the most commonly documented Elixhauser comorbidities in our population; the inclusion of this comorbidity may have resulted in over-adjustment; however, the incidence of chronic lung disease in our cohort is high so this would lead to a consistent result. Access to devices to utilize the required software for telemedicine visits may have influenced the patient’s choice of appointment type, and we were unable to quantify this effect. Furthermore, we studied a single center with unique patient and provider demographics; the results may not be generalizable. The study population largely involved those who identified as Hispanic white; the population in this study is representative of the Miami-Dade area, however, it is different from that of most other health systems in the United States. We only captured health care encounters within the University of Miami Health System; quantification of follow up visits, emergency department visits, and hospitalizations are limited by the inability to identify health care encounters that may have occurred in other health systems. If rates of care-seeking outside our system by patients seen via telemedicine versus in-person differed, and how any such difference may have impacted our results, is unknown; however, this limitation would not affect our primary outcome of CT scan orders.

Provider practice variation is a limiting factor as well, and we did not have sufficient documentation to determine if telemedicine was used primarily to reduce in person visits that the provider or patient may have seen as less useful. Provider preference and their experience with telemedicine may have further impacted the choice to utilize telemedicine. The primary outcome of association between CT scan orders and the usage of telemedicine may be affected by provider preference; however, the restriction of our cohort to include only patients seen by providers who saw patients in all years of the study (when telemedicine was and was not available) helps to mitigate this bias. Similarly, patient characteristics in different times of the study period may vary and influence the results; telemedicine was not utilized in our clinics until February of 2020 (supplemental Fig. 5). However, our sensitivity analysis limiting telemedicine to 2020–2021 and in-person visits to 2018–2019 aimed at minimizing the impact of selection bias in 2020–2021 (when both options were available) yielded similar results to our primary analysis.

The overlap of the Covid-19 pandemic with telemedicine use may have resulted in study limitations. Covid-19 test results were not available for outpatient visits; therefore, it is unclear whether telemedicine was utilized by choice or necessity for those who may have tested positive for Covid-19. Moreover, the impact of different peaks of the Covid-19 pandemic, when patients may have been more hesitant to seek in-person care and visit the ED, were not considered.

Finally, our study was limited by data availability. The majority of the sample did not have completed PFTs available in our EHR system, and therefore stratification based on lung function is limited. The low number of PFTs available is likely at least partially attributable to the covid-19 pandemic during which time PFTs were limited due to the desire to minimize aerosolization of the virus.

## Conclusions

We found that telemedicine in pulmonary clinics is associated with some increase in specific resource utilization. There was an increased odds of chest CT order with a concomitant decreased odds of chest x-ray order when patients were seen via telemedicine. While telemedicine usage was associated with a lower odds of ED visits and hospitalization, overall contact with the health care system was increased through tasks that may create a greater time burden for outpatient clinicians. As this clinic modality continues, unexpected downstream effects are likely. Caution and further study are warranted to determine how telemedicine can best serve patients and providers and which patient populations may benefit most from its use.

### Electronic supplementary material

Below is the link to the electronic supplementary material.

Supplementary Material 1

## Data Availability

All data generated or analyzed during this study are included in this published article and its supplementary information files.

## Abbreviations

COPD: Chronic Obstructive Pulmonary Disease
ED: Emergency Department
CT: Computed Tomography
PFT: Pulmonary Function Test
EHR: Electronic Health Record
ABG: Arterial Blood Gas
CF: Cystic Fibrosis
ILD: Interstitial Lung Disease
PH: Pulmonary Hypertension
FEV1: median Forced Expiratory Volume in one second
FVC: Forced Vital Capacity
DLCO: Diffusing Capacity of the Lungs for Carbon monoxide
RV: Residual Volume
CXR: Chest X-ray
Echo: Echocardiogram
Tele: Telemedicine
aOR: Adjusted Odds Ratio
