# Supplementary material for: Telemedicine and Resource Utilization in Pulmonary Clinic
Source: BMC Pulm Med. 2024 Jun 5;24:267. doi: 10.1186/s12890-024-03066-x (PMC11151562; doi:10.1186/s12890-024-03066-x)
Supplement: Supplementary file 1 — Supplementary Material 1 [file 12890_2024_3066_MOESM1_ESM.docx]

**Telemedicine and Associated Resource Utilization Patterns**

*Online Supplement*

Supplemental Figure 1. Flow Diagram


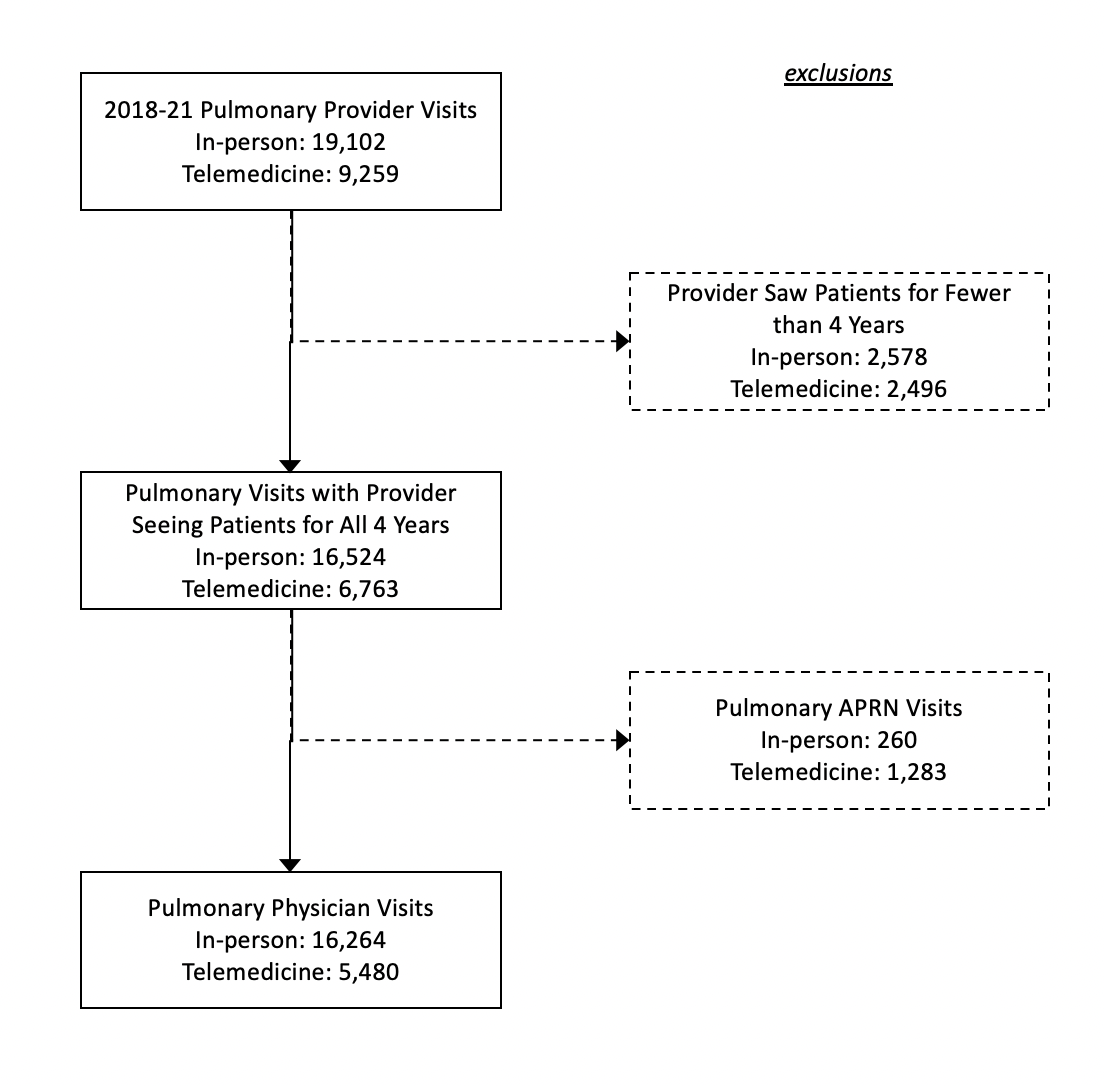


Flow diagram describing acquisition of final sample with inclusion and exclusion criteria

Supplemental Table 1. Diagnosis Grouping ICD-10 Codes

| Diagnosis | ICD-10 Codes |
| --- | --- |
| Abnormal imaging/Pulmonary Function Test/Arterial Blood Gas | E87.2, E87.4, R59.0, R79.81, R91.1, R91.8, R93.89, R94.2, R94.8, Z00.8, Z92.89 |
| Cystic Fibrosis and Congenital Disease | E84.0, E84.19, E84.8, E84.9, E88.01, E88.09, K86.81, P27.1, Q32.4, Q33.0, Q33.2, Q33.4, Q34.8, Z14.1, Z14.8 |
| Deconditioning and Dyspnea | G47.19, R06.00, R06.02, R06.03, R06.09, R06.4, R06.81, R06.89, R53.81, R53.83, Z91.89 |
| Hemo/Pneumothorax, Pleural Effusion, Pleuritis, and Trauma (Pleural Disease) | I50.1, J81.0, J81.1, J90, J91.8, J93.11, J93.12, J93.81, J93.83, J93.9, J94.0, J94.1, J94.2, J95.811, J98.19, J98.2, P25.1, R09.1, R84.0, S27.0XXS, S29.9XXA, S29.9XXD, S29.9XXS, T59.811A, T70.29XA, T79.7XXS, W16.92XS, Y93.15 |
| Hypoxemia, Respiratory Failure, Ventilation, Tracheostomy | J80, J95.02, J95.03, J96.00, J96.01, J96.02, J96.10, J96.11, J96.21, J96.22, J96.90, J96.91, J96.92, J98.6, R06.03, R09.02, Z43.0, Z93.0, Z99.11 |
| Interstitial Lung Disease and Pneumonitis | C83.39, C96.6, D71, D86.0, D86.1, D86.2, D86.89, D86.9, J12.9, J42, J61, J62.8, J66.8, J67.2, J67.8, J67.9, J68.0, J69.1, J70.0, J70.1, J70.3, J70.4, J84.01, J84.03, J84.10, J84.111, J84.112, J84.114, J84.115,, J84.116, J84.117, J84.170, J84.82, J84.89, J84.9, J98.4, J99, M05.10, M05.60, M30.1, M31.31, M31.7, M32.13, M33.21, M33.29, M33.91, M34.1, M34.81, M34.9, M35.02, M35.2, M35.9, Q85.1, R84.7, T46.2X5A, Z77.018, Z84.10 |
| Lung Nodules, Masses, and Malignancy | C34.00, C34.01, C34.02, C34.10, C34.11, C34.12, C34.2, C34.3, C34.31, C34.32, C34.81, C34.82, C34.90, C34.91, C34.92, C38.4, C45.0, C45.9, C7A.090, C7A.8, C77.1, C78.00, C78.01, C78.02, C78.1, C78.2, C85.99, D3A.00, D3A.090, D3A.8, J91.0, J92.0, J92.9, J94.8, J98.59, Q34.1, Q85.9, R22.2, R22.9, R59.0, R91.1, R91.8, Z77.090, Z12.2, Z77.29, Z85.110, Z85.118, Z85.89, Z87.898, Z90.2 |
| Obstructive Lung Disease | J41.0, J43.0, J43.1, J43.2, J43.8, J43.9, J44.0, J44.1, J44.9, J45.20, J45.21, J45.30, J45.31, J45.32, J45.40, J45.41, J45.42, J45.50, J45.51, J45.52, J45.901, J45.909, J45.990, J45.991, J45.998, J82.81, J82.83, J98.01, J98.9, O99.511, O99.513, P25.0, R94.2, Z51.81, Z68.3, Z79.51, Z87.09 |
| Pulmonary Hypertension and Cardiopulmonary Disease | B20, D75.9, I21.21, I27.0, I27.20, I27.21, I27.22, I27.23, I27.24, I27.29, I27.81, I27.89, I27.9, I28.0, I28.8, I37.0, I37.1, I97.89, K76.6, K76.81, M31.0, Q24.9, Q25.6, Q25.72, Q26.8, Z01.810 |
| Tobacco Use | F17.200, F17.201, F17.211, F17.219, F17.290, U07.0, Z71.6, Z72.0, Z72.89, Z77.22, Z87.891 |

Supplemental Table 2. Number of Patients Seen in Study Period with Number of Visits

| **# of Visits During Study Period** | **# of Patients** | **# of Visits** | **# (%) of Visits that were First Visits** |
| --- | --- | --- | --- |
| 1 | 4195 | 4195 | 4195 (100.0%) |
| 2 | 1877 | 3754 | 1877 (50.0%) |
| 3 | 968 | 2904 | 968 (33.3%) |
| 4 | 572 | 2288 | 572 (25.0%) |
| 5 | 334 | 1670 | 334 (20.0%) |
| 6 | 213 | 1278 | 213 (16.7%) |
| 7 | 159 | 1113 | 159 (14.3%) |
| 8 | 107 | 856 | 107 (12.5%) |
| 9 | 86 | 774 | 86 (11.1%) |
| 10 | 59 | 590 | 59 (10.0%) |
| 11 | 53 | 583 | 53 (9.1%) |
| 12 | 30 | 360 | 30 (8.3%) |
| 13 | 22 | 286 | 22 (7.7%) |
| 14 | 16 | 224 | 16 (7.1%) |
| 15 | 14 | 210 | 14 (6.7%) |
| 16 | 9 | 144 | 9 (6.3%) |
| 17 | 4 | 68 | 4 (5.9%) |
| 18 | 5 | 90 | 5 (5.6%) |
| 19 | 2 | 38 | 2 (5.3%) |
| 20 | 2 | 40 | 2 (5.0%) |
| 21 | 2 | 42 | 2 (4.8%) |
| 22 | 3 | 66 | 3 (4.5%) |
| 25 | 2 | 50 | 2 (4.0%) |
| 26 | 1 | 26 | 1 (3.8%) |
| 27 | 1 | 27 | 1 (3.7%) |
| 31 | 1 | 31 | 1 (3.2%) |
| 37 | 1 | 37 | 1 (2.7%) |

**Total number of patients: 8738**

Supplemental Figure 2: Proportion of Patients Seen Via Telemedicine
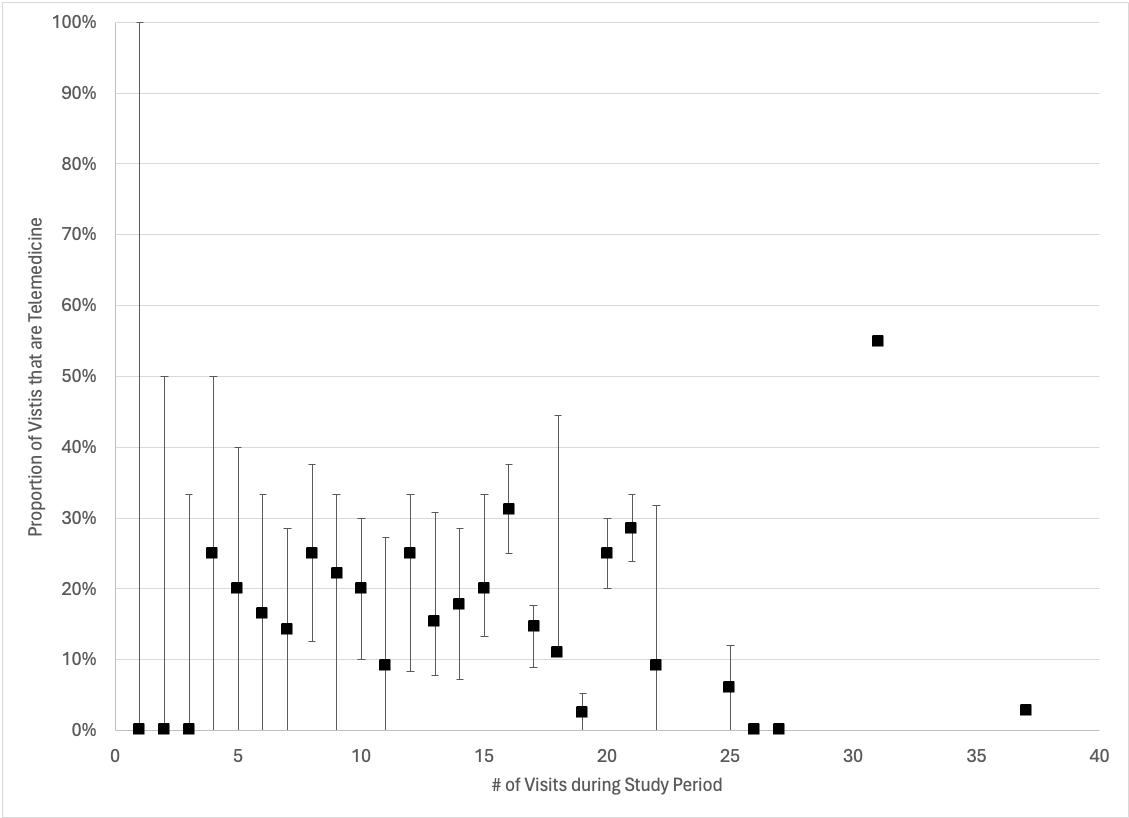


Proportion of patients seen via telemedicine based on number of visits during study period.

Supplemental Table 3. Individual Elixhauser Comorbidities

|  | In-Person Visits, N(%) | Telemedicine, N(%) | p-value |
| --- | --- | --- | --- |
| *Individual Comorbidities* |  |  |  |
| Congestive Heart Failure | 1,414 (8.7) | 356 (6.5) | <0.001 |
| Valvular Disease | 1,372 (8.4) | 341 (6.2) | <0.001 |
| Pulmonary Circulation Disorders | 965 (5.9) | 279 (5.1) | 0.020 |
| Peripheral Vascular Disease | 1,823 (11.2) | 530 (9.7) | 0.002 |
| Hypertension | 6,557 (40.3) | 1,924 (35.1) | <0.001 |
| Paralysis | 234 (1.4) | 54 (1.0) | 0.011 |
| Other Neurological Disorders | 1,978 (12.2) | 505 (9.2) | <0.001 |
| Chronic Lung Disease | 9,626 (59.2) | 3,040 (55.5) | <0.001 |
| Diabetes without Chronic Complications | 2,872 (17.7) | 724 (13.2) | <0.001 |
| Diabetes with Chronic Complications | 1,887 (11.6) | 447 (8.2) | <0.001 |
| Hypothyroidism | 2,359 (14.5) | 778 (14.2) | 0.58 |
| Renal Failure | 1,355 (8.3) | 305 (5.6) | <0.001 |
| Liver Disease | 2,256 (13.9) | 701 (12.8) | 0.044 |
| Peptic Ulcer Disease | 255 (1.6) | 96 (1.8) | 0.35 |
| AIDS/HIV | 171 (1.1) | 34 (0.6) | 0.004 |
| Lymphoma | 573 (3.5) | 171 (3.1) | 0.16 |
| Metastatic Cancer | 1,165 (7.2) | 489 (8.9) | <0.001 |
| Solid Tumor without Metastasis | 3,708 (22.8) | 1,426 (26.0) | <0.001 |
| Rheumatoid Arthritis | 2,168 (13.3) | 589 (10.8) | <0.001 |
| Coagulopathy | 1,112 (6.8) | 350 (6.4) | 0.25 |
| Obesity | 4,019 (24.7) | 1,137 (20.8) | <0.001 |
| Weight Loss | 2,852 (17.5) | 797 (14.5) | <0.001 |
| Fluid and Electrolyte Disorders | 2,633 (16.2) | 712 (13.0) | <0.001 |
| Blood Loss Anemia | 727 (4.5) | 247 (4.5) | 0.91 |
| Deficiency Anemia | 3,378 (20.6) | 1,027 (18.7) | 0.001 |
| Alcohol Abuse | 142 (0.9) | 45 (0.8) | 0.72 |
| Drug Abuse | 233 (1.4) | 77 (1.4) | 0.88 |
| Psychoses | 774 (4.8) | 207 (3.8) | 0.002 |
| Depression | 1,576 (9.7) | 491 (9.0) | 0.11 |

AIDS: acquired immunodeficiency syndrome; HIV: human immunodeficiency virus

Supplemental Table 4. Diagnosis Groupings

|  | Abnormal Imaging/  PFT/ABG | CF/  Congenital Disease | Deconditioning/  Dyspnea | Pleural Disease | Hypoxemia/RF/Vent/  Trach | ILD | Lung Nodules/Masses | Obstructive Lung Disease | pHTN/  Cardiopulmonary Disease | Tobacco Use |
| --- | --- | --- | --- | --- | --- | --- | --- | --- | --- | --- |
| Abnormal Imaging/PFT/ABG | 4859 | 14 | 384 | 66 | 63 | 228 | 3477 | 1310 | 55 | 182 |
| CF/Congenital Disease |  | 1057 | 18 | 2 | 29 | 4 | 10 | 68 | 2 | 1 |
| Deconditioning/  Dyspnea |  |  | 2614 | 44 | 100 | 242 | 333 | 579 | 170 | 50 |
| Pleural Disease |  |  |  | 341 | 8 | 23 | 64 | 58 | 17 | 11 |
| Hypoxemia/RF/  Vent/Trach |  |  |  |  | 529 | 78 | 54 | 172 | 36 | 8 |
| ILD |  |  |  |  |  | 3373 | 164 | 230 | 130 | 12 |
| Lung Nodules/Masses |  |  |  |  |  |  | 4012 | 1108 | 53 | 184 |
| Obstructive Lung Disease |  |  |  |  |  |  |  | 6705 | 107 | 204 |
| pHTN/  Cardiopulmonary Disease |  |  |  |  |  |  |  |  | 1362 | 4 |
| Tobacco Use |  |  |  |  |  |  |  |  |  | 425 |

ABG: arterial blood gas; PFT: pulmonary function test; CF: cystic fibrosis; RF: respiratory failure; Vent: ventilator associated; Trach: tracheostomy associated; ILD: interstitial lung disease; pHTN: pulmonary hypertension

Supplemental Figure 3. Telemedicine Use Stratified by Lung Pathology

A. Diagnosis*


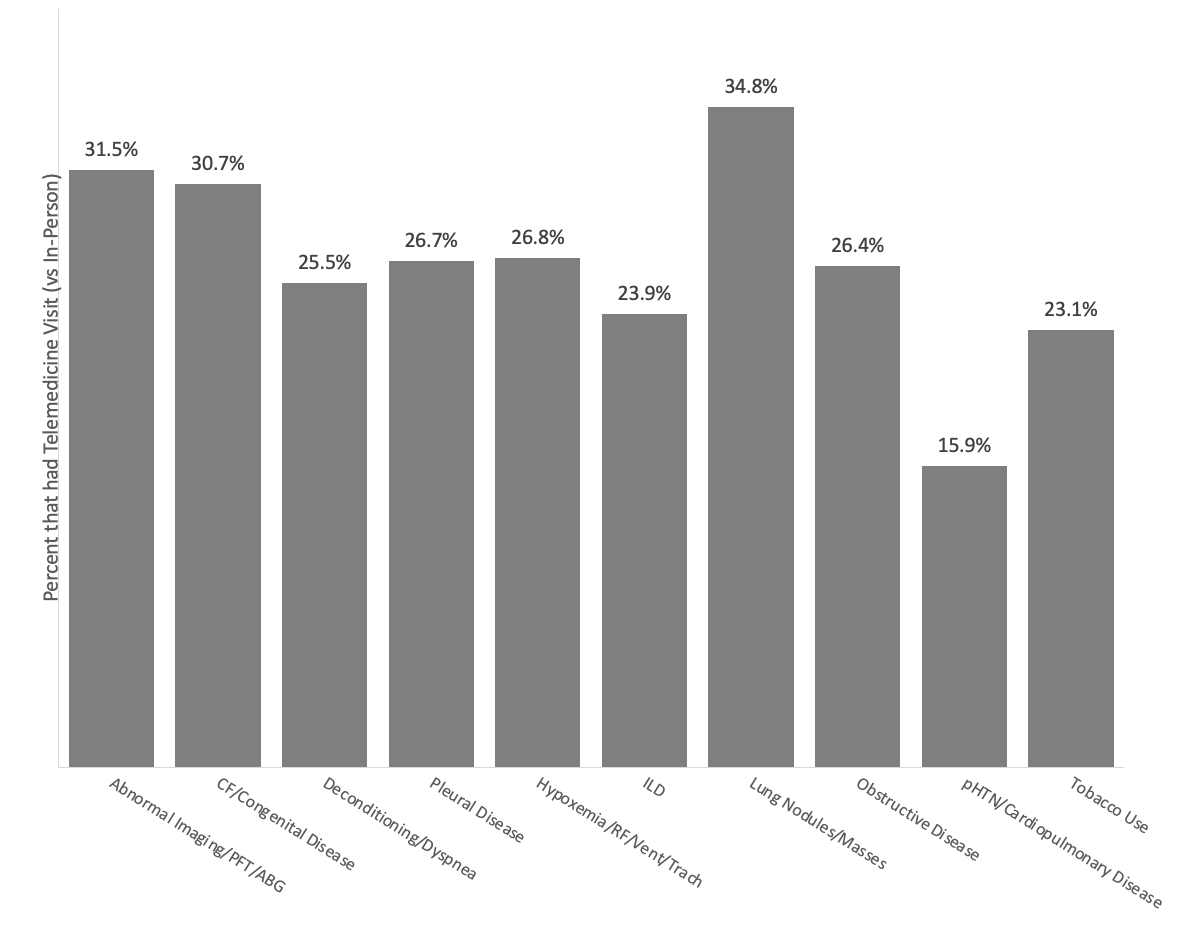


Bar graph displaying percentage of each diagnosis cohort that utilized telemedicine

B. Lung Function (FEV1)†


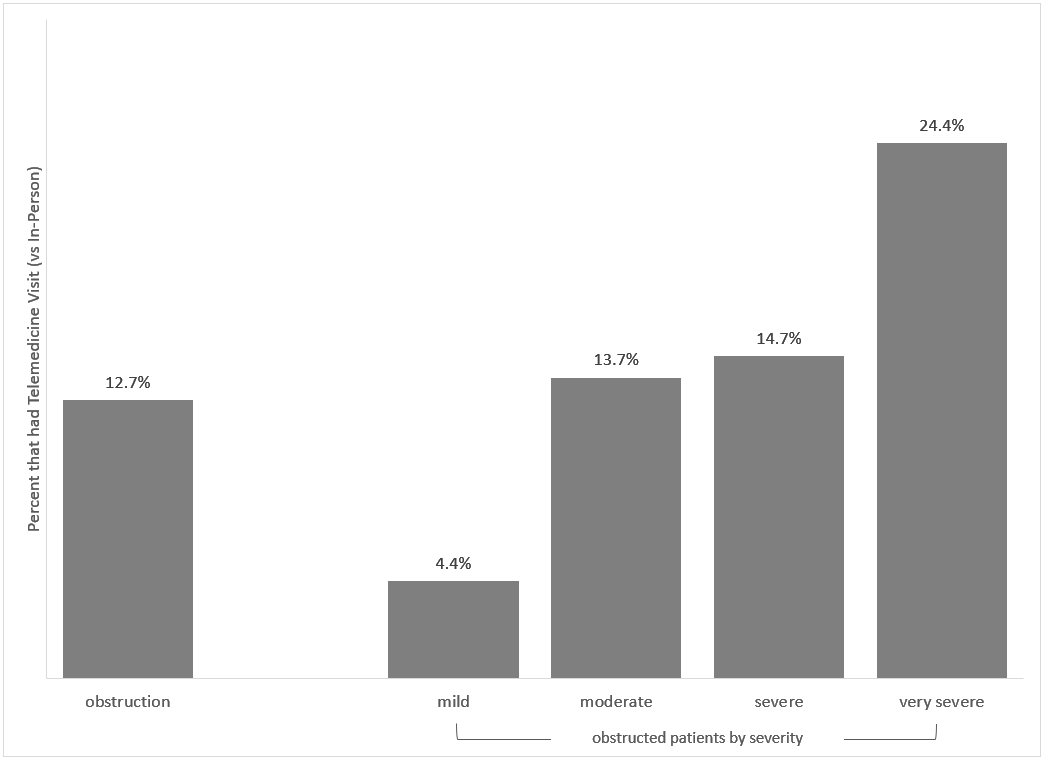


Bar graph displaying percent of patients with obstruction, and patients with different severities of obstruction who used telemedicine.

ABG: arterial blood gas; PFT: pulmonary function test; CF: cystic fibrosis; RF: respiratory failure; Vent: ventilator associated; Trach: tracheostomy associated; ILD: interstitial lung disease; pHTN: pulmonary hypertension

* Use of telemedicine higher (vs not having the diagnosis) for abnormal imaging/pft/abg (31.5% v 23.8%, p<0.001), cf/congenital disease (30.7% v 25.7%, p<0.001), lung nodules/masses (34.8% v 23.3%, p<0.001); use lower for ILD (23.9% v 26.5%, p=0.002), pHTN/cardioppulmonary disease (15.9% v 26.8%, p<0.001; all other p≥0.05

† One obstructed patient with unknown FEV not shown; p=0.006 across FEV1 categories

Supplemental Figure 4. Association of Telemedicine with Resource Use, Sensitivity Analysis with Cohort Limited to In-Person in 2018-19 vs Telemedicine in 2020-21.


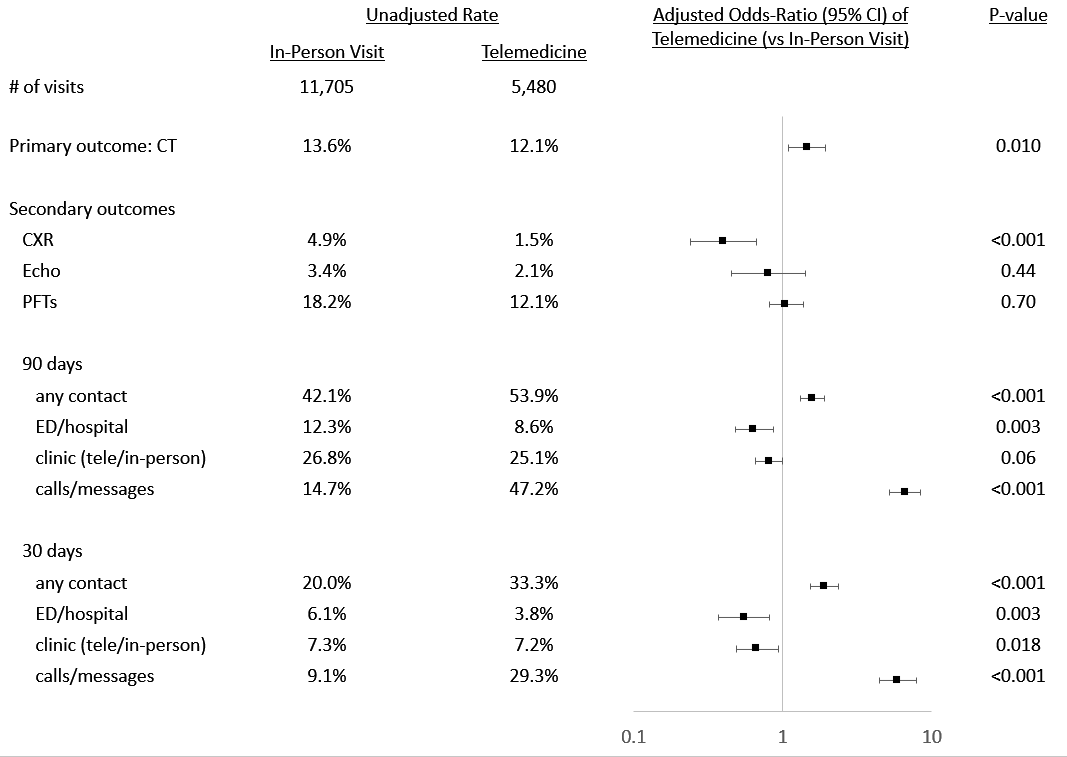


Figure showing unadjusted and adjusted odds ratios of outcomes when sensitivity analysis limiting the in-person cohort to 2018-19 and telemedicine cohort to 2020-21 was completed.

CT: chest computed tomography; CXR: chest x-ray; Echo: echocardiogram; PFT: pulmonary function test; ED: emergency department; tele: telemedicine

Supplemental Table 5: Association of Telemedicine with CT Use for ILD & OVD Patients (with PFTs)

|  | Unadjusted | | | Adjusted model | |
| --- | --- | --- | --- | --- | --- |
|  | *In-person* | *Teleclinic* |  |  |  |
|  | *% CT (# CT/# total)* | *% CT (# CT/# total)* | *p-value* | *OR (95% CI)* | *p-value* |
| *ILD* |  |  |  |  |  |
| Complete Cases with FVC | 41.7% (227/544) | 34.3% (24/70) | 0.23 | 0.86 (0.17,4.41) | 0.86 |
| Imputed FVC | 18.3% (471/2567) | 11.4% (92/806) | <0.001 | 1.41 (0.63,3.18) | 0.40 |
| *Obstructive disease* |  |  |  |  |  |
| Complete Cases with FEV1 | 21.8% (149/683) | 22.8% (33/145) | 0.80 | 1.84 (0.45,7.49) | 0.39 |
| Imputed FEV1 | 10.8% (531/4934) | 9.7% (171/1771) | 0.19 | 2.36 (1.30,4.27) | 0.005 |

CT: chest computed tomography; ILD: interstitial lung disease; OVD: obstructive disease; PFT: pulmonary function test; FVC: functional vital capacity; FEV1: forced expiratory volume in the first second;

Supplemental Figure 5: Proportion of Visits Conducted Via Telemedicine Over Time


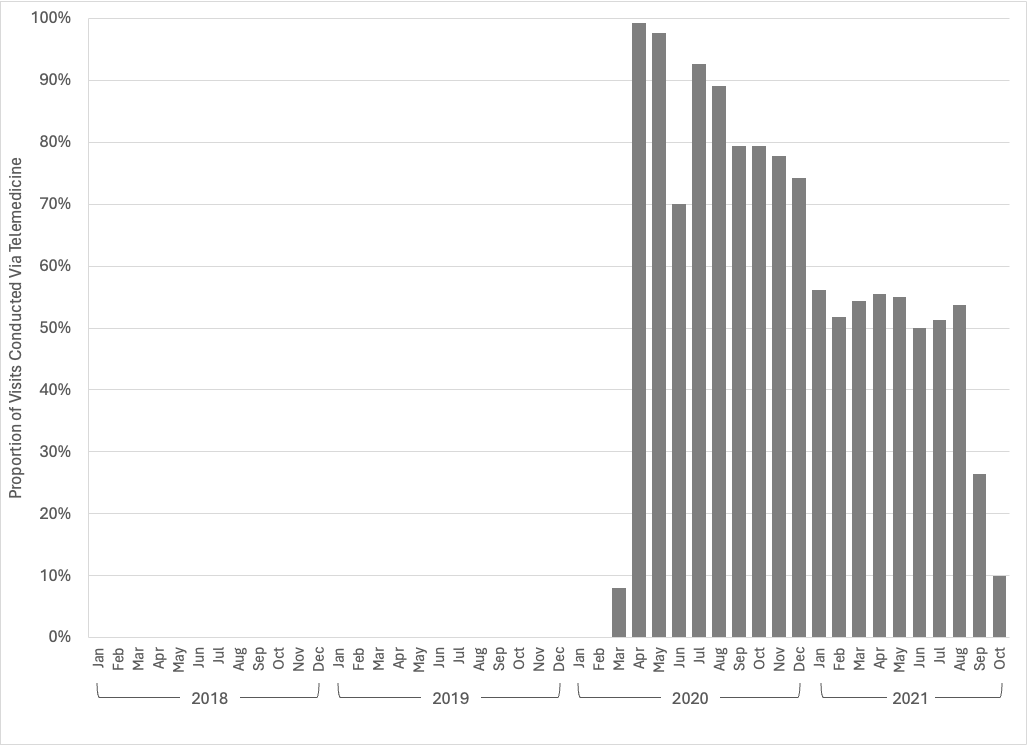


Figure showing proportion of visits conducted via telemedicine over time during the study period
